# Abnormal Anatomic Variation of Pancreaticobiliary Union in Magnetic Resonance Cholangiopancreatography Department of Radiology and Imaging in a Tertiary Care Centre: A Descriptive Cross-sectional Study

**DOI:** 10.31729/jnma.7944

**Published:** 2023-01-31

**Authors:** Sharma Paudel, Bidyanand Chaudhary, Pradeep Raj Regmi, Prakash Kayastha, Santosh Maharjan, Govinda Adhikari

**Affiliations:** 1 Department of Radiology and Imaging, Tribhuvan University Teaching Hospital, Maharajgunj, Kathmandu, Nepal; 2 Grande International Hospital, Kathmandu, Nepal

**Keywords:** *common bile duct*, *main pancreatic duct*, *magnetic resonance cholangiopancreatography*

## Abstract

**Introduction:**

The knowledge of the variations of the abnormal anatomy of pancreaticobiliary union is of great importance for understanding various pathologies of the biliary tract, gall bladder, and pancreas as well as to avoid surgical complications and morbidity which may arise from pancreaticobiliary maljunction. Moreover, it helps in the early diagnosis and preventive treatment of pancreaticobiliary disease. The objective of this study was to find out the prevalence of abnormal anatomic variations of the pancreaticobiliary union in magnetic resonance cholangiopancreatography examinations.

**Methods:**

This descriptive cross-sectional study was done in patients referred for Magnetic resonance cholangiopancreatography examinations for various clinical indications from 1 February 2021 to 30 May 2021. Ethical approval was obtained from the Institutional Review Committee [Reference number: 306 (6-11)E 2 077/078]. The variations in the pancreaticobiliary union, length of the common channel, and angle between the common bile duct and major pancreatic duct were obtained from the 1.5T magnetic resonance scanner in 90 patients. The three-dimensional magnetic resonance cholangiopancreaticography images were visually analyzed and classified into four categories. Convenience sampling method was used. Point estimate and 90% Confidence Interval were calculated.

**Results:**

Out of 90 patients, 73 (81.11%) (74.34-87.88, 90% Confidence Interval) patients had abnormal pancreaticobiliary union with pancreaticobiliary type as the most common occurrence seen in 33 (36.67%) patients.

**Conclusions:**

The prevalence of abnormal anatomic variation of pancreaticobiliary union was found to be higher than other studies done in similar settings.

## INTRODUCTION

Pancreaticobiliary maljunction (PBM) and its associated diseases are an area of interest in current radiology practice. The abnormal anatomical variations of pancreaticobiliary union (PBU) have become a common interest, especially in Asian countries. Intraoperative cholangiography and Endoscopic retrograde cholangiopancreatography (ERCP), although very accurate, are invasive methods for imaging the biliary tree and PBU along with the common channel.

Magnetic resonance cholangiopancreatography (MRCP) is an excellent non-invasive imaging technique for the visualization of the detailed anatomy of PBU. High-resolution cross-sectional, two-dimensional (2D), and three-dimensional (3D) projection images provide excellent anatomy which can be compared to ERCP and intraoperative cholangiograms.^1^

Accurate knowledge of normal anatomy of the distal end of the common bile duct and pancreatic duct that is pancreaticobiliary union has received attention because of its importance in pancreaticobiliary diseases.^2-4^ This could be the reference information for the clinical diagnosis of the pathological anatomy of PBM and its association with pancreaticobiliary disease.^5-7^

The objective of this study was to find out the prevalence of abnormal anatomic variations of the pancreaticobiliary union in magnetic resonance cholangiopancreatography examinations.

## METHODS

This descriptive cross-sectional study was performed in patients who were referred for MRCP examinations for various clinical indications to the Department of Radiology and Imaging, Tribhuvan University Teaching Hospital (TUTH), Maharajgunj, Nepal. Data were collected for a period of four months from 1 February 2021 to 30 May 2021 after receiving ethical approval from the Institutional Review Committee [Reference number: 306 (6-11)E 2 077/078]. Patients with a history of hepatic or biliary surgery and image with poor-quality with artifacts were excluded from the study. Informed consent forms were taken from the patients meeting the inclusion criteria. Convenience sampling method was used.

The sample size was calculated using the following formula:


$$\begin{array}{ll} \text{n} & ={\text{Z}}^{2}\times \frac{\text{p}\times \text{q}}{{\text{e}}^{\text{2}}}\\ & =1.64^{2}\times \frac{0.50\times 0.50}{0.10^{2}}\\ & =68 \end{array}$$

Where,

n = minimum required sample size
Z = 1.64 at 90% Confidence Interval (CI)
p = prevalence is taken as 50% for maximum sample size calculation
q = 1-p
e = margin of error, 10%

The minimum sample size calculated was 68. However, the final sample size taken was 90.

The Routine department protocol was followed for the MRCP examinations. MRCP was performed in 1.5T Magnetom Amira Siemens MRI scanner. The patients were thoroughly screened as per department guidelines for any ferromagnetic material. Detailed history was obtained from the patients before entering the scanner. Fresh pineapple juice was given to each patients 10-15 minute prior to the examination to reduce signal of fluid from the stomach. Patients were instructed to follow the instruction given by us at the time of scanning.

According to the anatomy of pancreaticobiliary ductal union based on our analysis, MRCP images were classified as normal type and abnormal type. The abnormal type was further divided into separate types, BP type and PB type. The length of the common channel was measured between the sphincter of Oddi and the junction of the pancreatic and bile duct. The angle between CBD and the major pancreatic duct was also obtained in degree. In our study, we have used the classification given by two different studies.^13^ The 3D SPACE images were reformatted with Maximum Intensity Projection (MIP). These images were then visually analyzed to determine the anatomic variation of the pancreaticobiliary union.

Data were entered and analysed using IBM SPSS version 20. Point estimate and 90% Confidence Interval were calculated.

## RESULTS

Out of 90 patients, 73 (81.11%) (74.34-87.88, 90% Confidence Interval) patients had abnormal pancreaticobiliary union. Among 90 patients, 17 (18.89%) patients had a normal pancreaticobiliary union (common channel length ≤8 mm). Out of 73 MRCP with abnormal pancreaticobiliary union, PB type was the most common type seen in 33 (36.67%) patients and BP type was the least commonly seen in 15 (16.66%) patients (Table 1).

**Table 1:** Descriptive statistics of different types of pancreaticobiliary union (n= 73).

Types of pancreaticobiliary junction		n (%)
Abnormal	Separate type	25 (27.78)
	PB type	33 (36.67)
	BP type	15 (16.66)

Among them 39 (43.33%) were males and 51 (56.67%) were female. The mean age was 49.17±16.33 years. Sixty-five (72.22%) patients had a common channel and 25 (27.78%) patients had no common channel. The mean length of the common channel was 9.53±3.24 mm with a minimum length of 3.90 mm and a maximum length of 23.0 mm. The mean angle between CBD and the major pancreatic duct was 56.34±21.56° (range 20° to 90°).

In PB type all angles were acute angled ranging from 21°-65°. Based on gender, pancreaticobiliary angle ranged from 20°-90° with an average of 48.53° in males and 23°-90° with an average of 59.81° in females.

The most common type of pancreaticobiliary union was PB type in both sexes (Table 2).

**Table 2:** Sex distribution of pancreaticobiliary union (n= 73).

Types of pancreaticobiliary union		Male	Female
Abnormal	Separate type	11 (15.07)	14 (19.18)
	PB type	17 (23.29)	16 (21.92)
	BP type	4 (5.48)	11 (15.07)

## DISCUSSION

In our study,out of 90 patients, 73 (81.11%) patientshad abnormal pancreaticobiliary union which is much higher compared to another study where abnormal variation was seen in 34.73%.^2^ PB type was the most common occurrence seen in 33 (36.67%) patients. The average length of the common channel was 9.53±3.24 mm (average 9.04 mm in males and 10.2 mm in females). The normal length of the common channel for this study was assigned as ≤8 mm. Our study showed the maximum length of the common channel in PB type was 16.3 mm and BP type was 23 mm which was also the maximum length of the common channel. There was no significant difference in the length of the common channel according to gender.

Our study showed that all the PB types were acute angled but 33.33% was only right-angled in the BP type. In a similar study done in Kosovo, it was revealed that PB type was equal to an acute angle whereas BP type was equal to a right angle. In the same way, BP type (31.70%) was most common in this study which is different from our study where PB type (36.67%) is most common.^1^ This variation can be due to the difference in sample size or the difference in demographic population. In their study, the average angle between CBD major pancreatic duct was 35.6° (SD±21.10°) which was comparatively less than our study i.e. 56.33° (SD±21.55°). This may be due to the small sample size. In their study they found one BP type making right-angled between CBD and the major pancreatic duct while we found five such cases of the same type. In their study, the mean length of the common channel was 4.5 mm with a maximum 15 mm but in our study, we found a mean length of 9.53±3.24 mm with a maximum 23 mm.

The junction of the common bile duct (CBD) and pancreatic duct outside the wall of the duodenum that forms the common channel (>8 mm) is called an abnormal pancreaticobiliary junction (APBJ).^2,4^ There is still no unified recognition of the normal length of the common duct. The length of the common channel varies. Some authors suggest 8 mm or longer as a long common channel,^4^ however, others suggest longer than 15 mm.^5^

Currently, MRI is considered to be the method of choice for the anatomic study of the biliary system owing to its high sensitivity, non-invasive or atraumatic nature with the absence of ionizing radiation. Due to several technical improvements introduced in its protocol over the years, MRCP allows excellent visualization of the biliary system with an important role in the early diagnosis and treatment of pancreaticobiliary disease as well as important information regarding the anatomy of pancreaticobiliary duct union.

The junction of the pancreatic duct and bile duct is crucial for sphincteric control of pancreatic and bile juice drainage with bidirectional regurgitation that occurs if the union is above the sphincter of Oddi.^3^ The joining of the ducts was classified into four categories: Normal type, V or separate type, B-P type and P-B type.^2^ Most of the studies have proved that the P-B type was equal to the acute angle and the B-P type was equal to the right angle.^4,7^ According to Kimura's classification the joining of CBD and the pancreatic duct was categorized into two types: type I in which the pancreatic duct enters the CBD and type II in which CBD enters the pancreatic duct.^5^ In 2015, the Committee on Diagnostic Criteria of the Japanese Study Group on Pancreaticobiliary Maljunction (PBM) also proposed a classification into four different categories: (A) stenotic type (B) nonstenotic type (C) dilated channel type and (D) complex type.^7^ The frequency of anomalous arrangement of the pancreaticobiliary duct ranged from 1.5-3.2%.^8^ Different authors have studied Pancreaticobiliary Junction Union and its clinical significance.^9-13^ In our study we have used the classification based on other studies.^1,3^

In another study done in 694 cases showed the average length of the common channel was 7.9 mm with 241 (34.70% ) anomalous unions which were 84 (12.1%) PB type, 85 (12.2%) BP type and 72 (10.4%) separate type.^2^ In our study, the average length of the common channel was 9.53±3.24 mm which was similar to this study. But, the anomalous union in our study was 81.11%, PB type 36.67% being the most common similar to this study. The variation in the average value of types of anomalous was due to differences in sample size. In another study, it was reported that the mean length of the common channel was 4.7±2.5 mm (range 1.6-18.4 mm) among 63% of 102 normal ERCP which was half of the finding in our study.^4^ This variation could be due to the difference in the modality of examination (ERCP vs MRCP) and the small sample size. The length of the common channel and angle between CBD and major pancreatic duct was measured because they have clinical aid in pre and post-surgical complications and morbidity as well as help in providing a reference for differential diagnosis of pancreaticobiliary diseases.

The main limitation of the study was the small sample size and small demographic area. Functional MRCP i.e. contrast (lipophilic) or secretin-stimulated MRCP was not done which would make it easier to depict the union of CBD and major pancreatic duct as secretin dilates the pancreatic duct. The measurements were taken in one shot without averaging so it might have caused some measurement errors.

## CONCLUSIONS

The prevalence of abnormal variations of the pancreaticobiliary union was found to be higher than in other studies done in similar settings, probably because of the sample size, demographic as well as geographic differences. Detailed, accurate pre-operative identification of pancreaticobiliary anatomical variants is essential to avoid severe post-surgical morbidity and complications. Radiologists and hepatobiliary surgeons have to be aware of the possibility of PBM in patients undergoing MRCP examination. Patients with PBM can be given preventive treatment in cases with recurrent pancreatitis and biliary inflammation.
